# 558. Cirrhosis Drives Higher Mortality and Prolonged Hospitalization in Burn Patients

**DOI:** 10.1093/jbcr/irag033.298

**Published:** 2026-04-06

**Authors:** Mare G Kaulakis, Hilary Liu, Joshua Yoon, Christopher J Fedor, Sarah M Tepe, Alain C Corcos, Francesco M Egro

**Affiliations:** University of Pittsburgh Medical Center, Mercy Burn Center, Pittsburgh, PA; University of Pittsburgh Medical Center, Pittsburgh, PA; University of Pittsburgh Medical Center, Pittsburgh, PA; University of Pittsburgh Medical Center, Department of Plastic Surgery, Pittsburgh, PA; University of Pittsburgh Medical Center, Pittsburgh, PA; University of Pittsburgh Medical Center, Pittsburgh, PA; University of Pittsburgh Medical Center, Pittsburgh, PA

## Abstract

**Introduction:**

Cirrhosis impairs liver function, coagulation, and immunity, potentially worsening burn outcomes. However, its impact on hospitalization and mortality remains unclear. This study evaluated cirrhosis and outcomes in burn patients using a national dataset.

**Methods:**

A retrospective review was performed using data from the Burn Care Quality Platform from 2013 to 2022. Patients with cirrhosis were compared to those without. Extracted data included demographics, burn characteristics, and hospital course. Univariate and multivariable regressions assessed associations with mortality, LOS, liver failure, and graft/flap loss. Models adjusted for age, TBSA, mechanism, comorbidities, and burn depth.

**Results:**

Of 263 623 patients, 1134 (0.4%) had cirrhosis. They were older (57.6 vs. 37.2 yrs, *p*<.001), more often male (71.5% vs. 66.2%, *p*=.001), and had higher rates of alcohol use (32.7% vs. 4.9%), smoking (44.1% vs. 23.8%), diabetes (29.2% vs. 11.5%), CHF (12.4% vs. 2.5%), and HTN (44.9% vs. 20.4%) (all *p*<.001). Burns were larger (9.8% vs. 7.5% TBSA, *p*<.001), with longer injury-to-admission time (4.3 vs. 2.8 days, *p*=.001). Flame (38.0% vs. 31.9%) and flash burns (10.8% vs. 8.6%) were more common, while scalds were less frequent (15.3% vs. 33.0%, *p*<.001). Cirrhotics underwent fewer procedures (0.55 vs. 0.72, *p*<.001), but graft/flap loss did not differ (0.66% vs. 0.33%, *p*=.318).

Unadjusted outcomes showed markedly worse prognosis. Mortality was higher (18.8% vs. 3.1%, *p*<.001), as were LOS (14.8 vs. 9.2 days), ICU stay (9.3 vs. 5.7), infection (5.4% vs. 2.3%), and liver failure (0.37% vs. 0.02%) (all *p*<.001). On adjusted analysis, cirrhosis independently predicted mortality (OR 6.53, 95% CI 5.17–7.92), liver failure (OR 7.68, 95% CI 2.56–12.78), and longer LOS (β = 1.15 days, *p*<.01). No independent association was found with infection (OR 1.10, *p*=.564) or graft/flap loss (OR 1.00, *p*=.996).

Subgroup analysis showed that cirrhosis was independently associated with mortality across all TBSA categories (*p*<.001). No significant interaction with TBSA or burn etiology was observed, suggesting cirrhosis confers risk independent of injury extent or mechanism.

**Conclusions:**

Cirrhosis independently predicts mortality, prolonged hospitalization, and liver failure in burn patients regardless of burn size or cause. These findings highlight the need for heightened vigilance and tailored management in this high-risk group. Future work should explore targeted interventions and the role of early multidisciplinary care for cirrhotics.

**Applicability of Research to Practice:**

This study underscores the importance of accounting for cirrhosis as a critical risk factor in burn research and highlights the need for stratification by liver disease status in clinical practice and research.

**Funding for the study:**

N/A.

